# Variability of Root Traits in Spring Wheat Germplasm

**DOI:** 10.1371/journal.pone.0100317

**Published:** 2014-06-19

**Authors:** Sruthi Narayanan, Amita Mohan, Kulvinder S. Gill, P. V. Vara Prasad

**Affiliations:** 1 Department of Agronomy, Kansas State University, Manhattan, Kansas, United States of America; 2 Department of Crop and Soil Sciences, Washington State University, Pullman, Washington, United States of America; National Institute of Plant Genome Research, India

## Abstract

Root traits influence the amount of water and nutrient absorption, and are important for maintaining crop yield under drought conditions. The objectives of this research were to characterize variability of root traits among spring wheat genotypes and determine whether root traits are related to shoot traits (plant height, tiller number per plant, shoot dry weight, and coleoptile length), regions of origin, and market classes. Plants were grown in 150-cm columns for 61 days in a greenhouse under optimal growth conditions. Rooting depth, root dry weight, root: shoot ratio, and shoot traits were determined for 297 genotypes of the germplasm, Cultivated Wheat Collection (CWC). The remaining root traits such as total root length and surface area were measured for a subset of 30 genotypes selected based on rooting depth. Significant genetic variability was observed for root traits among spring wheat genotypes in CWC germplasm or its subset. Genotypes Sonora and Currawa were ranked high, and genotype Vandal was ranked low for most root traits. A positive relationship (R^2^≥0.35) was found between root and shoot dry weights within the CWC germplasm and between total root surface area and tiller number; total root surface area and shoot dry weight; and total root length and coleoptile length within the subset. No correlations were found between plant height and most root traits within the CWC germplasm or its subset. Region of origin had significant impact on rooting depth in the CWC germplasm. Wheat genotypes collected from Australia, Mediterranean, and west Asia had greater rooting depth than those from south Asia, Latin America, Mexico, and Canada. Soft wheat had greater rooting depth than hard wheat in the CWC germplasm. The genetic variability identified in this research for root traits can be exploited to improve drought tolerance and/or resource capture in wheat.

## Introduction

Wheat (*Triticum* spp.) is one of the most important food crops in the world in terms of the area harvested, production, and productivity [1]. Wheat is grown in a wide variety of environments from tropical to temperate. Although wheat has a wide range of climatic adaptability, its productivity is limited by several abiotic stresses. Among those stresses, drought is the most widespread limitation to wheat productivity under dry-land conditions. Consequently, developing drought-tolerant wheat genotypes has been the focus of many wheat improvement programs. Root traits are critical for soil exploration and water and nutrient uptake, and are important for crop improvement under drought conditions [2], [3].

The effectiveness of a deep root system in maintaining yield under drought conditions has been confirmed by simulation studies across several years and environments in the USA [4]. A deep root system helps the plant to avoid drought stress by extracting water stored in deep soil layers (reviewed by [5] and [6]). Total root length was associated with drought tolerance in wheat because it affects the distribution of roots in the soil and influences the amount of water uptake [7]. Increased root diameter was associated with drought tolerance in rice (*Oryza sativa* L.) because thicker roots have large xylem vessels with increased axial conductance and are more efficient in penetrating deep soil layers to extract water [8], [9]. Root length density (RLD) increases the prolificacy of the root system, and was the most important trait for increased phosphorus uptake in wheat [10]. Root length density in the active root zone (0–30 cm soil depth) was correlated with water and nutrient uptake and yield under water-sufficient and water-limited conditions in chick pea (*Cicer arietinum* L.) [11], [12], [13]. Root dry weight and root: shoot ratio were positively correlated with drought tolerance in rice [14]. Fine root production in response to soil drying contributed to drought tolerance in turf grass (*Festuca arundinacea* Schreb.) [15]. Fine roots increase water and nutrient absorption because they increase root surface area per unit mass [16]. Fine roots constitute the major component of the root systems and are the most active part of the root system in extracting water and nutrients [17], [18], [19].

Despite the importance of root traits in drought tolerance, little work has been done to include drought-adaptive root traits in breeding for drought-tolerant wheat varieties. Most wheat improvement programs have concentrated on above-ground components, particularly for decreasing plant height and increasing harvest index. Crop breeding programs have largely ignored root traits, mainly because of the difficulties associated with root recovery and evaluating root traits in situ. In addition, large phenotypic plasticity of root traits in response to changes in soil conditions, and lack of high-throughput and cost-effective screening techniques make root studies highly challenging [2], [20], [21]. As a result, limited information is available on genetic variability of root traits in wheat. Exploring genetic variability of root traits could assist wheat improvement programs in developing varieties with desired root traits for drought tolerance or target environments. An understanding of the relationship of root traits to the shoot traits that contribute to grain yield is also essential to achieve improvements in productivity.

The region of origin of crop plants has implications in plant breeding as they act as potential centers to locate useful genes. Region of origin may provide useful sites for germplasm exploration to identify traits that improve productivity [22]. The adaptation profiles of domesticated plants well reflect their region of origin [23]. The agro-climatic conditions of specific regions might influence the evolution of adaptive root traits in crop plants. However, the influence of region of origin on root traits is not investigated in wheat.

Based on kernel hardness and color, wheat genotypes can be classified into different market classes. Suitability of each market class to a location depends largely on rainfall, temperature, and soil conditions. Recent findings suggested that market classes of wheat differed for coleoptile length and effect of coleoptile length on seedling emergence [24]. This suggests that differences may exist among market classes for traits contributing to productivity. However, no studies have investigated the differences in root traits among different market classes of wheat.

The objectives of this research were to (i) characterize variability of root traits among spring wheat genotypes, (ii) determine whether root traits are related to plant height, shoot dry weight, tiller number per plant, and coleoptile length, and (iii) determine whether the regions of origin and market classes of genotypes have any influence on root traits.

## Materials and Methods

### Germplasm

The germplasm used in this study was Cultivated Wheat Collection (CWC) [24] consisting of 297 spring wheat genotypes (Table S1) released since 1901. The germplasm was developed using the seed material obtained from the Germplasm Resources Information Network (GRIN), International Maize and Wheat Improvement Center (CIMMYT), and Washington State University Historical Collection. These genotypes represent cultivars from 27 different countries (Table S1); Egypt (2), Libya (1), Lebanon (1), Armenia (1), Turkey (3), Iraq (1), Jordan (1), USA (190), Canada (14), Mexico (31), India (6), Pakistan (8), Nepal (2), Bangladesh (1), Australia (9), Argentina (6), Chile (2), Brazil (1), Colombia (3), Guatemala (1), Paraguay (4), Uruguay (1), Russia (2), Kenya (1), South Africa (1), Japan (1), and Germany (1). Two genotypes were not confirmed of their origin. The genotypes from USA represent the most popular cultivars during each 5-year interval since 1950 from all major breeding programs in the country. Genotypes in CWC germplasm also represented four different market classes of wheat: soft white spring (SWS), soft red spring (SRS), hard white spring (HWS), and hard red spring (HRS) (Table S1).

### Experimental Details

This research was conducted in controlled environment facilities (greenhouse) at the Department of Agronomy, Kansas State University, Manhattan, KS. Two independent experiments (2011 and 2012) were conducted to evaluate the variability of root traits among spring wheat genotypes. The greenhouse was equipped with an automated sulfur vaporizer (Rosemania, Franklin, TN) that vaporized sulfur for 1 h between 23∶00 and 24∶00 h. Sulfur vaporization was done from the start of the experiment as a preventive measure against powdery mildew. Plants were grown in PVC columns with inside diameter of 7.5 cm and height of 150 cm. The columns had plastic caps at the bottom with a central hole of 0.5 cm diameter for drainage. Rooting medium was Turface MVP (PROFILE Products LLC, Buffalo Grove, IL), which had a bulk density of 576.66±32 kg m^−3^. Turface is calcined, non-swelling illite and silica clay, and allows easy separation of roots. The rooting medium was fertilized with Osmocote (Scotts, Marysville, OH), a controlled-release fertilizer with 19∶6∶12 N:P_2_O_5_:K_2_O, respectively, at 4 g per column before sowing. A systemic insecticide, Marathon 1% G (a.i.: Imidacloprid: 1–[(6–Chloro–3–pyridinyl)methyl]–N–nitro–2–imidazolidinimine; OHP, Inc., Mainland, PA) was applied at 1 g per column before sowing to control sucking pests. Twenty seeds of each genotype were weighed before sowing to estimate seed size (individual seed weight). Three seeds of a single genotype were sown at 4 cm depth in each column on 28 December 2011 and 6 December 2012. After emergence, plants were thinned to one plant per column, which was maintained until harvest. Plants were irrigated daily (0.9±0.1 L per day) through an automated drip irrigation system until harvest to avoid water stress. Emissions from drip-tubes were examined weekly for proper water delivery. Irrigation was provided three times per day at 06∶00, 12∶00, and 18∶00 h. Plants were maintained under optimum temperature (24/14°C, daytime maximum/nighttime minimum) conditions from sowing to harvest at a photoperiod of 16 h. The fungicide, Bumper 41.8 EC (a.i.: Propiconazole: 1–[[2–(2,4 dichlorophenyl)–4–propyl–1,3–dioxolan–2–yl]Methyl]–1H–1,2,4–triazole; 1.2 mL L^−1^; Makhteshim Agan of North America, Inc., Raleigh, NC) was also applied at 20 d after sowing to prevent powdery mildew. The insecticide and fungicide treatments helped to maintain the plants without any pest or pathogen problems until harvest. Plants were harvested at 61 d after sowing when more than 50% of the population reached flowering stage.

### Data Collection

Shoot traits measured in this study were plant height, number of tillers per plant, shoot dry weight, and coleoptile length (see the measurement details below). These traits were measured on all 297 genotypes in the CWC germplasm. Height, tiller number, and growth stage of all plants were recorded 1 d before harvest. Plant height was determined as the distance between Turface level and the last leaf ligule. At harvest, the PVC columns were gently inverted at about 140° to let the contents of columns (Turface and plants with the entire root system) slip down to the ground. Roots were carefully separated from the Turface without any breakage in the root system. The shoot of each plant was separated by cutting at the base of the stem. After removing shoots, roots were laid on a flat surface and stretched to measure their length (from the base of the stem to the tip of the root system) as an estimate of rooting depth. Rooting depth was measured using the above procedure for all 297 genotypes in the CWC germplasm. The root system was then washed, placed between moist paper towels, sealed in Ziploc bags (S.C. Johnson & Sons, Inc. Racine, WI), transported to the laboratory, and stored at 4°C. Fifteen genotypes that were ranked the highest and 15 genotypes that were ranked the lowest for rooting depth were selected for further complete root analyses. This subset of 30 genotypes represented cultivars from Australia, Turkey, USA, Mexico, and Canada. The subset included genotypes representing the four market classes, SWS, SRS, HWS, and HRS. Root system of each of these 30 genotypes was stretched and sliced into 30-cm long portions. Each portion was submerged in a water bath (20 cm×15 cm×2 cm) to maximize separation of roots and to minimize their overlap, and scanned using an Epson photo scanner (Epson Perfection V700 with 6400 dpi resolution) (Epson, Long Beach, CA). Images of scanned roots of the 30 genotypes were analyzed using WinRHIZO Pro image analysis system (Regent Instruments, Inc., Quebec City, QC) to estimate total root length (sum of the lengths of all roots in the root system), total root surface area, root volume, average root diameter, length, surface area and RLD of roots in 0–30 cm soil depth, fine root (roots with diameter <0.25 mm) length, fine root surface area, and fine root volume [25], [26]. Root length density in each 30-cm depth of root system was calculated as the ratio of root length to the volume of 30-cm section of the PVC column, and it represented RLD in each 30 cm of soil depth [22]. After scanning, root systems were packed in paper bags for drying. Roots and shoots of all 297 genotypes were dried to constant weight at 60°C for determining dry weight. Root: shoot ratio for each of the 297 genotypes was calculated as the ratio of root dry weight to shoot dry weight.

Coleoptile length was measured according to the procedure of [24]. Fifteen uniform-sized seeds of each of the 297 genotypes with no physical damage were placed in the middle of a moist germination paper (Heavy Germination paper #SD 7615L; Anchor Paper Co., Saint Paul, MN), about 1 cm apart with the germ end down. The germination paper was then folded vertically in half with the seed placed in the crease, and the folded half was again folded horizontally four times and placed in a plastic tray with holes at the base to drain excess water. The plastic trays were then placed inside a completely darkened box and kept in a growth chamber at a constant temperature of 22°C. After 10 d, the coleoptile length of 10 randomly-selected seedlings of each genotype was recorded to the nearest millimeter measuring from the base of the seed to the tip of the coleoptile.

### Statistical Analyses

The experimental design was a randomized complete block in 2011 (Experiment 1) and 2012 (Experiment 2) for the greenhouse studies. There were two blocks (replications) in both years. Analysis of variance was performed on genotypes using the GLM procedure in SAS (Version 9.2, SAS Institute) for root and shoot traits. The probability threshold level (α) was 0.05. Genotype was treated as a fixed effect, and replication nested within year was treated as a random effect. Genotype, replication, and year were used as class variables. Separation of means was done using the LSD test (P<0.05). The CORR procedure in SAS was used to find out the correlation between different root and shoot traits. Pearson correlation coefficient was used as a measure of degree of correlation between root and shoot traits. The REG procedure in SAS was used to regress root traits against shoot traits.

## Results

### Genetic Variability of Root and Shoot Traits

**Figure 1:**
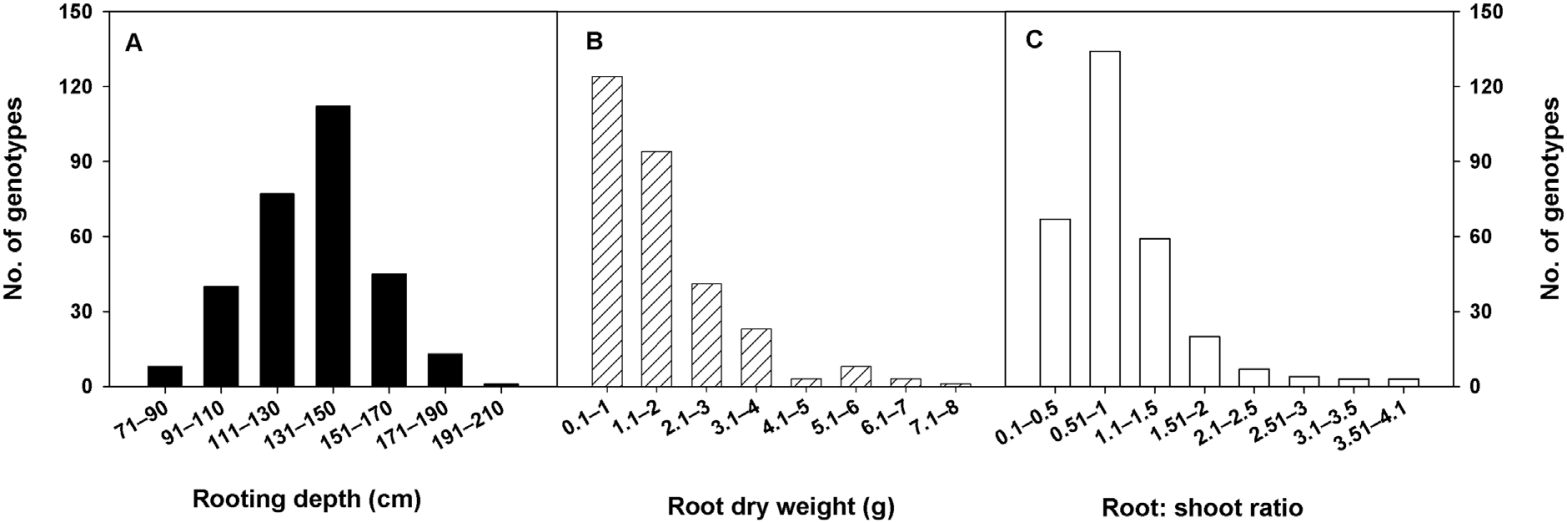
Distribution of rooting depth, root dry weight, and root: shoot ratio among 297 spring wheat genotypes of the Cultivated Wheat Collection.

**Figure 2:**
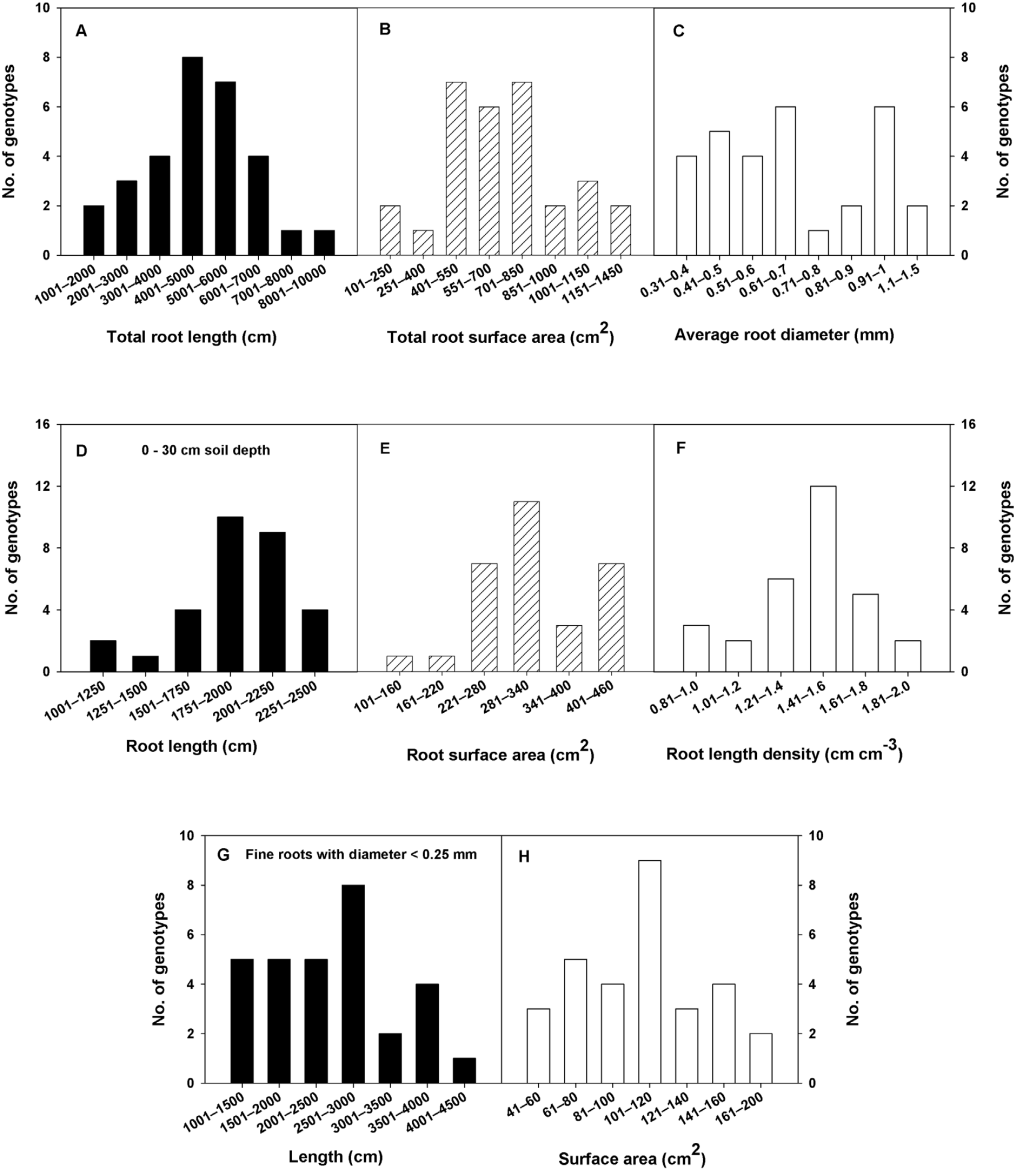
Distribution of major root traits within the subset of 30 spring wheat genotypes. The represented traits were measured for the 15 deepest and 15 smallest roots. Root length density is the ratio of root length in 0–30 cm depth of root system to the volume of the 30-cm section of the PVC column.

Significant variability was observed for root and shoot traits among spring wheat genotypes in the CWC germplasm or its subset (Table 1). Because there was no significant interaction between genotype and year for most of the traits, data were pooled across years. More than 100% variation was observed between minimum and maximum values of all root traits (Table 1). Range of major root traits was 77–202 cm, rooting depth and 0.23–7.6 g, root dry weight in the CWC germplasm and 1692–9094 cm, total root length and 184–1435 cm^2^, total root surface area in the subset (Table 1). Extent of variability for different root traits among genotypes in the CWC germplasm and the subset is shown in Fig. 1 and 2, respectively. Ranking of genotypes based on the numerical values of different root traits in the CWC germplasm and the subset are given in Table 2 and 3, respectively. Genotypes Sonora and Currawa had increased rooting depth (Table 2), total root length, total root surface area, average root diameter, fine root length, and fine root surface area (Table 3). Similarly, genotypes Vandal and Marquis had decreased rooting depth (Table 2), total root length, total root surface area, average root diameter, fine root length, and fine root surface area (Table 3). Genotypes Sonora and Currawa were also ranked high and genotype Vandal was also ranked low for total root length, total root surface area, and RLD in the 0–30 cm depth of soil (Table 3). Genotype Florence Aka Quality had increased rooting depth and root dry weight (Table 2). Genotypes Federation 67 and McVEY had decreased root diameter, but increased root length and RLD in 0–30 cm depth of soil (Table 3).

**Table 1:** Analyses of variance results on effects of year (Y), genotype (G), and Y×G interaction and range for various root and shoot traits.

Traits†	df (G)‡	P values			Range
		Y	G	Y*G	
Rooting depth (cm)	296	<.0001	<.0001	0.1003	77–202
Plant height (cm)	296	<.0001	<.0001	<.0001	11–60
Shoot dry weight (g)	296	<.0001	0.0098	0.2849	0.17–6.2
Root dry weight (g)	296	<.0001	<.0001	0.2263	0.23–7.6
Root: shoot ratio	296	<.0001	0.0341	0.0567	0.18–4.1
Tiller number per plant	296	0.2104	<.0001	0.9986	1–14
Total root length (cm)	29	<.0001	0.0412	0.0834	1692–9094
Total root surface area (cm^2^)	29	<.0001	0.0034	0.0545	184–1435
Root volume (cm^3^)	29	<.0001	0.0021	0.0064	1.6–18
Average root diameter (cm)	29	<.0001	<.0001	0.0641	0.35–1.4
**Root traits in 0–30 cm soil depth**					
Length (cm)	29	<.0001	0.0089	0.0501	1166–2484
Surface area (cm^2^)	29	<.0001	0.0027	0.1907	144–447
Root length density (cm cm^−3^)	29	<.0001	0.0082	0.0501	0.857–1.83
**Traits of fine roots with diameter <0.25 mm**					
Length (cm)	29	<.0001	0.0463	0.1454	1005–4540
Surface area (cm^2^)	29	<.0001	0.0455	0.1196	41–195
Volume (cm^3^)	29	0.0020	0.0275	0.0852	0.16–0.76

† Rooting depth, plant height, shoot dry weight, root dry weight, root: shoot ratio, and tiller number were estimated for all the 297 genotypes of the Cultivated Wheat Collection. Other traits were estimated only within the subset of 30 genotypes.

‡ Degrees of freedom for genotype.

**Table 2:** Spring wheat genotypes that were ranked high and low for rooting depth, root dry weight, and root: shoot ratio in the Cultivated Wheat Collection of 297 genotypes.

	Rooting depth (cm)	Root dry weight (g)	Root: shoot ratio
Highest 10†	Florence Aka Quality (202±26)‡ ^a^	YSCA-1 (7.6±5.9)^a^	Olaf (4.1±2.2)^a^
	Sonora (190±19)^a^	Florence Aka Quality (6.6±4.6)^a^	Whitebird (4.0±3.3)^a^
	Marfed (186±24)^a^	Wilbur (6.1±2.2)^a^	Ramona 50 (3.9±2.9)^ab^
	Idaho 61M3404 (183±43)^a^	Schlanstedt (6.1±4.2)^a^	Schlanstedt (3.4±2.4)^abc^
	Lemhi 66 (183±16)^a^	Challis (5.8±3.5)^a^	Redchaff (3.4±1.9)^abc^
	Union (183±8)^a^	Kenya Kwale (5.5±4.1)^a^	Kenya Kwale (3.1±1.8)^abc^
	Walladay (182±31)^a^	Kinney (5.5±3.7)^a^	Eden (3.0±1.6)^abc^
	Sakha 69 (180±25)^a^	Utac (5.4±3.1)^a^	Faislabad 83 (2.9±2.5)^abc^
	Currawa (179±17)^a^	Pacific Bluestem (5.32.7)^a^	WA 7175 (2.8±2.1)^abc^
	Lemhi (176±20)^a^	Pirsabak 85 (5.1±2.3)^a^	Wells (2.3±1.6)^bc^
Lowest 10	Vandal (96±30)^b^	ND 66 (0.35±0.10)^b^	White Marquis (0.27±0.015)^d^
	MN 6616M (93±18)^b^	ND 22 (0.35±0.15)^b^	White Federation (0.26±0.09)^d^
	Era (89±17)^b^	Faislabad 83 (0.35±0.15)^b^	Era (0.26±0.17)^d^
	Cumhuriyet 75 (87±18)^b^	Wells (0.34±0.19)^b^	Scarlet (0.24±0.10)^d^
	Yecora Rojo (85±27)^b^	MN 6616M (0.33±0.15)^b^	Vanna (0.22±0.07)^d^
	Marquis (84±25)^b^	Era (0.33±0.15)^b^	Ceres (0.22±0.09)^d^
	Sonora 64 (84±18)^b^	Vandal (0.32±0.12)^b^	Peak (0.22±0.09)^d^
	ND 287 (81±16)^b^	ND 287 (0.25±0.03)^b^	Sonora 64 (0.19±0.09)^d^
	Baw898 (78±15)^b^	Sonora 64 (0.23±0.11)^b^	McKay (0.19±0.05)^d^
	Hope (77±6)^b^	Calidad (0.23±0.09)^b^	Wadual (0.18±0.11)^d^
LSD	46	2.7	1.6

† Genotypes were ranked based on the numerical values of root traits.

‡ Values in parentheses are means ± standard errors of the respective traits. Values followed by different letters are significantly different according to a LSD test at P<0.05.

**Table 3:** Ranking of spring wheat genotypes for major root traits within the subset of 30 genotypes.

	Total root length (cm)	Total root surface area (cm^2^)	Average root diameter (mm)	Fine roots with diameter less than 0.25 mm		Root traits in 0–30 cm soil depth		
				Length (cm)	Surface area (cm^2^)	Length (cm)	Surface area (cm^2^)	Root length density (cm cm^−3^)
Highest 10†	Sonora (9094±1762)‡ ^a^	CI014953 (1435±571)^a^	Onas (1.41±0.42)^a^	Sonora (4540±1100)^a^	Sonora (195±34)^a^	Sel 90 (2484±151)^a^	Pilcraw (447±36)^a^	Sel 90 (1.83±0.02)^a^
	Currawa (7249±1446)^ab^	Sonora (1386±297)^ab^	CI014953 (1.05±0.21)^ab^	Currawa (3823±967)^ab^	Currawa (164±29)^ab^	WA 6101 (2471±35)^a^	Hybrid 123 (438±70)^ab^	WA 6101 (1.82±0.01)^a^
	Pilcraw (6785±921)^abc^	Pilcraw (1099±135)^abc^	Awned Onas (1.03±0.38)^abc^	WA 6101 (3694±1516)^abc^	WA 6101 (153±54)^abc^	II-58–60 (2356±218)^ab^	CI014953 (437±91)^ab^	II-58–60 (1.73±0.03)^ab^
	Hyper (6649±2415)^abc^	Onas (1054±230)^abcd^	Pilcraw (1.01±0.30)^bcd^	Marfed (3586±1091)^abcd^	Marfed (150±39)^abcd^	McVEY (2307±392)^abc^	Currawa (432±38)^abc^	McVEY (1.7±0.06)^abc^
	WA 6101 (6364±2180)^abcd^	Currawa (1039±166)^abcde^	Sonora (0.97±0.28)^bcd^	Hyper (3583±1489)^abcd^	Hyper (149±55)^abcd^	Sonora (2221±119)^abcd^	WA 6101 (423±50)^abcd^	Sonora (1.63±0.01)^abcd^
	Marfed (6133±1799)^abcde^	Hyper (909±292)^abcdef^	Hybrid 123 (0.97±0.27)^bcd^	Lemhi 66 (3303±1200)^abcde^	Lemhi 66 (141±44)^abcde^	Federation 67 (2215±286)^abcd^	Onas (419±46)^abcd^	Federation 67 (1.63±0.03)^abcd^
	Lemhi 66 (5970±2041)^abcde^	Union (878±211)^bcdefg^	Currawa (0.97±0.32)^bcd^	Pilcraw (3083±523)^abcdef^	Pilcraw (138±14)^abcde^	PITIC62 (2205±190)^abcd^	Sonora (415±45)^abcd^	PITIC62 (1.62±0.06)^abcd^
	Onas (5848±1586)^abcde^	Awned Onas (834±191)^bcdefg^	Union (0.93±0.26)^bcde^	Union (2995±409)^abcdefg^	Union (134±16)^abcde^	Pilcraw (2141±85)^abcde^	Hyper (376±44)^abcde^	Pilcraw (1.57±0.07)^abcde^
	Union (5844±707)^abcde^	WA 6101 (831±229)^edefg^	Kitt (0.86±0.22)^bcdef^	Andes-56 (2846±1081)^abcdefgh^	Awned Onas (121±15)^abcdef^	Currawa (2115±241)^abcde^	Awned Onas (344±47)^abcdef^	Currawa (1.55±0.02)^abcde^
	CI014953 (5824±1961)^abcde^	Lemhi 66(775±260)^cdefg^	Sel 90 (0.81±0.23)^bcdefg^	Awned Onas (2753±466)^abcdefgh^	Onas (120±36)^abcdef^	Hyper (2082±157)^abcde^	Peak 72 (343±93)^abcdef^	Hyper (1.53±0.01)^abcde^
Intermediate 10	Awned Onas (5531±817)^abcde^	Andes-56 (771±377)^cdefg^	Peak 72 (0.74±0.27)^bcdefgh^	CI014953 (2633±978)^abcdefgh^	Andes-56 (117±38)^abcdef^	Awned Onas (2066±155)^abcde^	Union (340±68)^abcdef^	Awned Onas (1.52±0.01)^abcde^
	Andes-56 (5262±2029)^bcde^	Marfed (762±218)^cdefgh^	Hyper (0.68±0.21)^bcdefgh^	Fielder (2605±931)^abcdefgh^	CI014953 (115±37)^abcdef^	Marfed (2055±209)^abcde^	Kitt (338±33)^abcdef^	Marfed (1.51±0.02)^abcde^
	Federation 67 (5065±3065)^bcdef^	Hybrid 123 (736±164)^cdefghi^	Marfed (0.67±0.15)^bcdefgh^	Federation 67 (2577±1583)^bcdefgh^	Fielder (110±36)^bcdef^	Marquis (2030±471)^abcde^	Federation 67 (335±54)^abcdef^	Marquis (1.49±0.05)^abcde^
	McVEY (4729±2527)^bcdef^	Conley (735±379)^cdefghi^	Langdon (0.66±0.15)^bcdefgh^	Onas (2550±684)^bcdefgh^	Federation 67 (109±63)^bcdef^	Kitt (1989±149)^abcdef^	Sel 90 (329±22)^abcdef^	Kitt (1.46±0.02)^abcdef^
	Copper (4672±1936)^bcdef^	Langdon (688±376)^cdefghi^	PITIC62 (0.64±0.15)^cdefgh^	McVEY (2536±1341)^bcdefgh^	McVEY (105±51)^bcdef^	Fielder (1976±458)^abcdef^	McVEY (322±65)^abcdef^	Fielder (1.45±0.04)^abcdef^
	Fielder (4539±1714)^bcdef^	Federation 67 (671±377)^cdefghi^	WA 6101 (0.64±0.13)^cdefgh^	Copper (2445±1001)^bcdefgh^	Sel 90 (103±33)^bcdef^	Peak 72 (1971±218)^abcdef^	Penjamo T 62 (308±64)^abcdef^	Peak 72 (1.45±0.02)^abcdef^
	Conley (4500±2285)^bcdef^	McVEY (636±359)^cdefghi^	Lemhi (0.61±0.21)^defgh^	Zak (2363±685)^bcdefgh^	Copper (102±37)^bcdef^	Zak (1969±210)^abcdef^	II-58–60 (306±44)^abcdef^	Zak (1.45±0.02)^abcdef^
	Zak (4485±1311)^bcdef^	Copper (620±297)^cdefghi^	II-58–60 (0.56±0.19)^efgh^	II-58–60 (2224±535)^bcdefgh^	Zak (101±23)^bcdef^	Hybrid 123 (1927±168)^abcdef^	PITIC62 (305±27)^abcdef^	Hybrid 123 (1.42±0.02)^abcdef^
	Saunders (4319±2719)^bcdef^	Saunders (617±446)^cdefghi^	Penjamo T 62 (0.54±0.11)^efgh^	Sel 90 (2196±583)^bcdefgh^	II-58–60 (97±21)^bcdef^	Union (1916±193)^abcdefg^	Lemhi 66 (294±54)^bcdef^	Union (1.41±0.02)^abcdefg^
	Sel 90 (4091±1056)^bcdef^	Zak (606±206)^cdefghi^	Zak (0.52±0.11)^fgh^	Saunders (2065±1109)^bcdefgh^	Saunders (88±13)^bcdef^	Penjamo T 62 (1906±201)^abcdefg^	Marfed (290±43)^cdef^	Penjamo T 62 (1.40±0.06)^abcdefg^
Lowest 10	Hybrid 123 (4012±713)^bcdef^	Fielder (536±221)^defghi^	Andes-56 (0.52±0.09)^fgh^	Conley (1940±1063)^bcdefgh^	Conley (84±42)^bcdef^	Calidad (1848±499)^abcdef^	Andes-56 (288±77)^defg^	Calidad (1.36±0.13)^abcdefg^
	II-58–60 (4000±889)^bcdef^	Sel 90 (530±135)^defghi^	Copper (0.48±0.09)^fgh^	Penjamo T 62 (1866±810)^cdefgh^	Hybrid 123 (83±13)^cdef^	Saunders (1811±478)^abcdefgh^	Fielder (279±92)^defg^	Saunders (1.33±0.05) ^abcdefgh^
	Langdon (3468±1304)^cdef^	Peak 72 (490±222)^efghi^	Fielder (0.47±0.14)^fgh^	Hybrid 123 (1852±280)^cdefgh^	Penjamo T 62 (79±30)^cdef^	Andes-56 (1769±250)^bcdefgh^	Zak (262±28)^efg^	Andes-56 (1.30±0.04)^bcdefgh^
	Penjamo T 62 (3291±1265)^cdef^	II-58–60 (476±92)^fghi^	Conley (0.47±0.06)^fgh^	PITIC62 (1783±312)^cdefgh^	PITIC62 (79±13)^cdef^	Lemhi 66 (1698±152)^bcdefgh^	Saunders (260±97)^efg^	Lemhi 66 (1.25±0.02) ^bcdefgh^
	PITIC62 (3290±489)^cdef^	Kitt (464±81)^fghi^	Federation 67 (0.45±0.03)^gh^	Marquis (1734±839)^defgh^	Marquis (72±29)^def^	Copper (1660±289)^cdefgh^	Langdon (259±108)^efg^	Copper (1.22±0.06)^cdefgh^
	Kitt (2935±500)^def^	Penjamo T 62 (445±147)^fghi^	McVEY (0.41±0.02)^h^	Langdon (1486±548)^efgh^	Kitt (66±14)^ef^	Onas (1619±390)^defgh^	Conley (248±94)^efg^	Onas (1.19±0.03)^defgh^
	Marquis (2909±1335)^def^	PITIC62 (428±60)^fghi^	Saunders (0.39±0.04)^h^	Kitt (1365±191)^fghe^	Langdon (63±20)^ef^	CI014953 (1528±336)^efgh^	Copper (242±80)^efg^	CI014953 (1.12±0.05)^efgh^
	Peak 72 (2552±764)^ef^	Marquis (327±151)^ghi^	Vandal (0.36±0.02)^h^	Peak 72 (1175±222)^fgh^	Peak 72 (52±11)^f^	Vandal (1323±341)^fgh^	Marquis (242±68)^efg^	Vandal (0.97±0.05)^fgh^
	Calidad (1848±499)^f^	Calidad (210±66)^hi^	Marquis (0.36±0.01)^h^	Calidad (1083±287)^gh^	Calidad (45±9)^f^	Langdon (1246±320)^gh^	Calidad (210±66)^fg^	Langdon (0.92±0.03)^gh^
	Vandal (1692±650)^f^	Vandal (184±68)^i^	Calidad (0.35±0.02)^h^	Vandal (1005±413)^h^	Vandal (41±14)^f^	Conley (1166277)^h^	Vandal (144±34)^g^	Conley (0.86±0.03)^h^
LSD	3605	553	0.39	1950	80	673	144	0.47

† Genotypes were ranked based on the numerical values of root traits.

‡ Values in parentheses are means of the respective traits. Values followed by different letters are significantly different according to a LSD test at P<0.05.

### Relationship between Root and Shoot Traits

**Figure 3:**
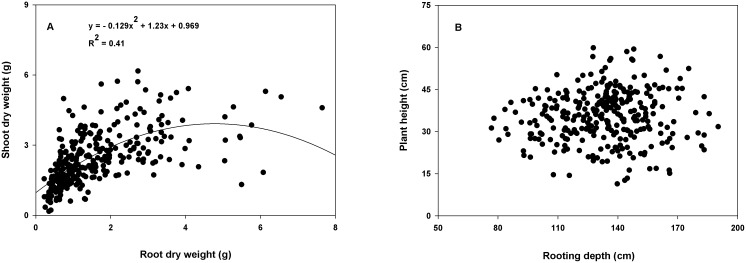
Relationship between root and shoot traits of 297 spring wheat genotypes of the Cultivated Wheat Collection. Slope of the regression line was not significant in Fig. B.

**Figure 4:**
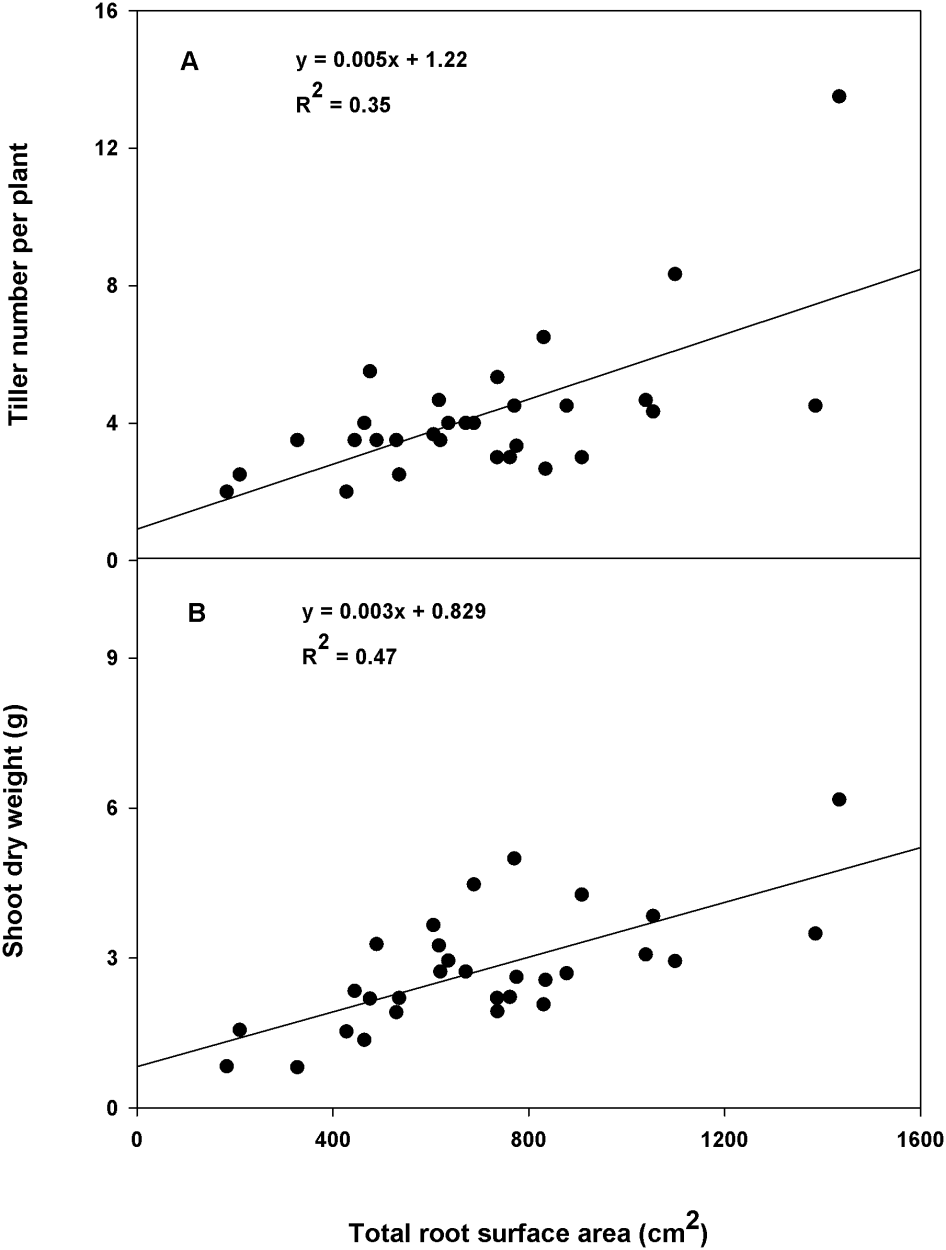
Relationship of total root surface area with tiller number per plant and shoot dry weight within the subset of 30 spring wheat genotypes.

**Figure 5:**
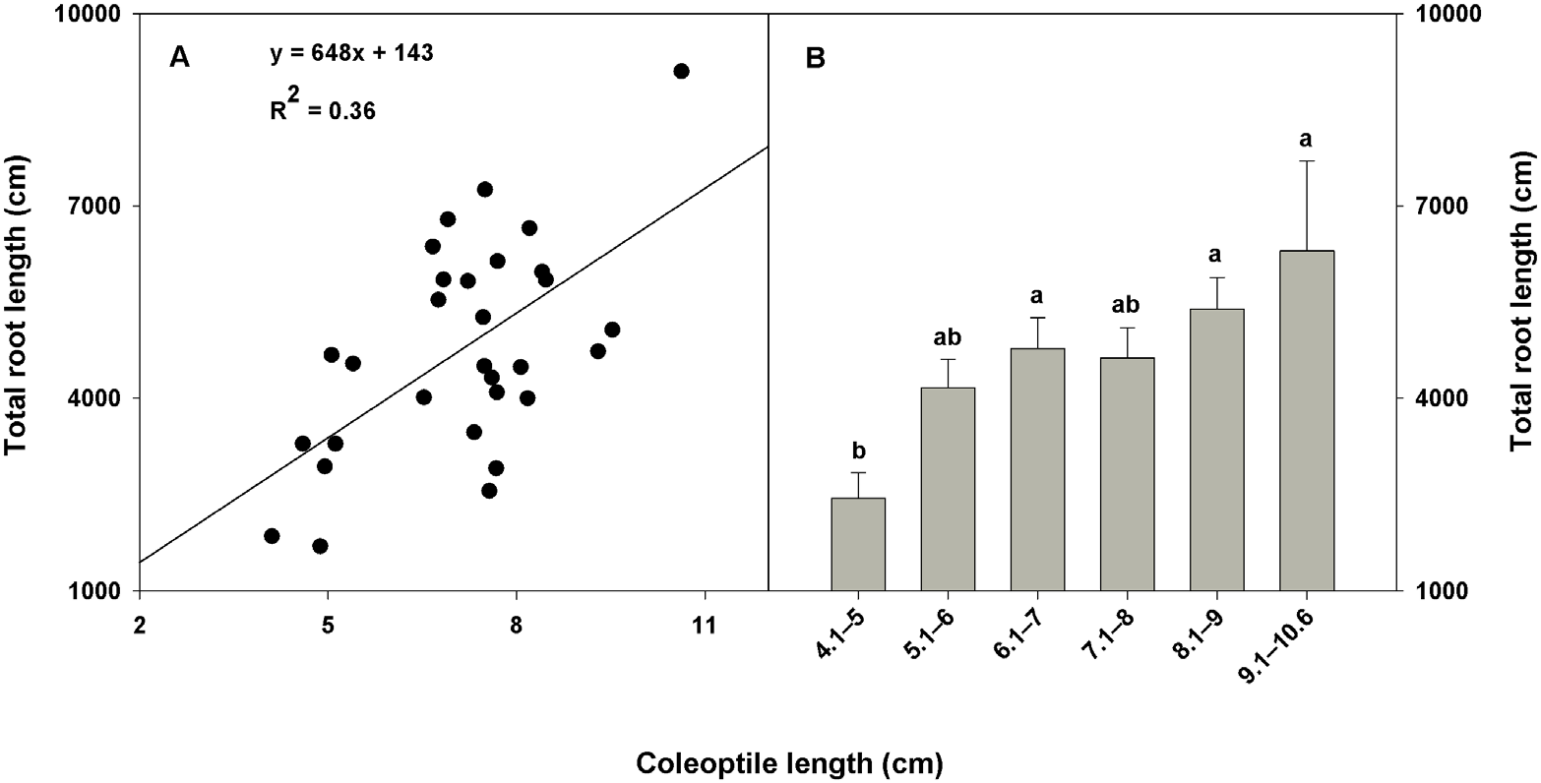
Relationship between coleoptile length and total root length within the subset of 30 spring wheat genotypes. Error bars (Fig. B) represent standard errors.

A positive relationship (coefficient of determination [R^2^]≥0.35) was found between root dry weight and shoot dry weight within the CWC germplasm (Fig. 3A) and between total root surface area and tiller number (Fig. 4A); total root surface area and shoot dry weight (Fig. 4B); and total root length and coleoptile length within the subset (Fig. 5A). A correlation coefficient of >0.50 was observed for the correlation of shoot dry weight with root dry weight and rooting depth within the CWC germplasm and shoot dry weight with total root surface area, and root volume; tiller number with total root surface area and root volume; and coleoptile length with total root length and total root surface area within the subset (Table 4, 5). Slope of the regression between plant height and rooting depth was not significant within the CWC germplasm (Fig. 3B). In addition, plant height did not show correlation with most root traits within the CWC germplasm (Table 4) or the subset (Table 5). Coleoptile length had a significant effect on total root length within the subset (P<0.001; Fig. 5B). Genotypes with longer coleoptiles (>8 cm) had significantly greater total root length than genotypes with shorter coleoptiles (≤5 cm; Fig. 5B).

**Table 4:** Correlations among various root and shoot traits of 297 spring wheat genotypes of the Cultivated Wheat Collection.

Traits	Root dry weight	Shoot dry weight	Root: shoot ratio	Plant height	Tiller number	Coleoptile length	Seed size
Rooting depth	0.43†****‡	0.51****	−0.13*	NS§	0.28****	0.16**	NS
Root dry weight		0.57****	0.33****	NS	0.46****	0.14*	NS
Shoot dry weight			−0.18**	0.36****	0.55****	0.13*	NS
Root: shoot ratio				−0.35****	NS	NS	NS
Plant height					NS	NS	NS
Tiller number						0.17**	NS
Coleoptile length							NS

† Values in each cell represent Pearson correlation coefficient.

‡ *, **, ***, and **** indicate significance at 0.05, 0.01, 0.001, and 0.0001 probability levels, respectively.

§ Not significant at 0.05 probability level.

**Table 5:** Correlations among various root and shoot traits within the subset of 30 spring wheat genotypes.

Traits	Total rootsurface area	Root volume	Average rootdiameter	Rootingdepth	Root dryweight	Shoot dryweight	Root: shootratio	Plant height	Tiller number	Coleoptile length	Seed size
Total root length	0.88†****‡	0.73****	0.51**	0.79****	0.59***	0.47**	NS§	NS	NS	0.66***	NS
Total root surface area		0.94****	0.70****	0.68****	0.87****	0.69****	NS	NS	0.61***	0.51**	NS
Root volume			0.78****	0.60***	0.81****	0.69****	NS	NS	0.54**	0.43*	NS
Average root diameter				0.55**	0.57***	NS	NS	NS	0.38*	NS	NS

† Values in each cell represent Pearson correlation coefficient.

‡ *, **, ***, and **** indicate significance at 0.05, 0.01, 0.001, and 0.0001 probability levels, respectively.

§ Not significant at 0.05 probability level.

### Region of Origin, Market Class, and Root Traits of Wheat Genotypes

**Figure 6:**
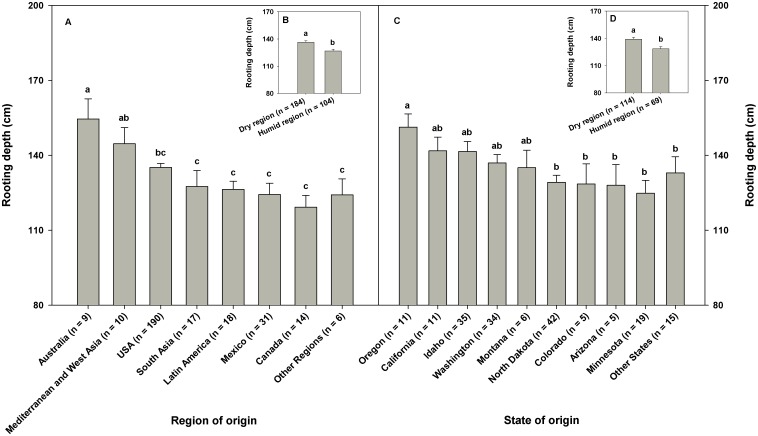
Rooting depth of spring wheat genotypes originating from different wheat growing regions in the world (Fig. A), and different states in the USA (Fig. C). Fig. A represents 295 genotypes of the Cultivated Wheat Collection (297 genotypes) because two genotypes were not confirmed of their origin. Fig. C represents 183 of the 190 genotypes originated from USA because seven genotypes were not confirmed of their state of origin. Mediterranean – Egypt, Libya, and Lebanon; West Asia – Armenia, Turkey, Iraq, and Jordan; South Asia – India, Pakistan, Nepal, and Bangladesh; Latin America – Argentina, Chile, Brazil, Colombia, Guatemala, Paraguay, and Uruguay; Other Regions – Russia, Japan, Germany, Kenya and South Africa. Other States – Indiana, Nebraska, Nevada, Oklahoma, South Dakota, Utah, Vermont, and Wisconsin. Dry region in Fig. B included Argentina, Armenia, Australia, Chile, Egypt, Germany, Iraq, Japan, Jordan, Kenya, Lebanon, Libya, Mexico, Pakistan, South Africa, Turkey, and USA states of Arizona, California, Colorado, Idaho, Montana, Nebraska, Nevada, Oregon, Utah, and Washington. Similarly, humid region in Fig. B included Bangladesh, Brazil, Canada, Colombia, Guatemala, India, Nepal, Paraguay, Russia, Uruguay, and USA states of Indiana, Minnesota, North Dakota, Oklahoma, South Dakota, Vermont, and Wisconsin. Two genotypes with unknown country of origin and seven genotypes with unknown states of origin in the USA were not included in Fig. B. Error bars represent standard errors.

**Figure 7:**
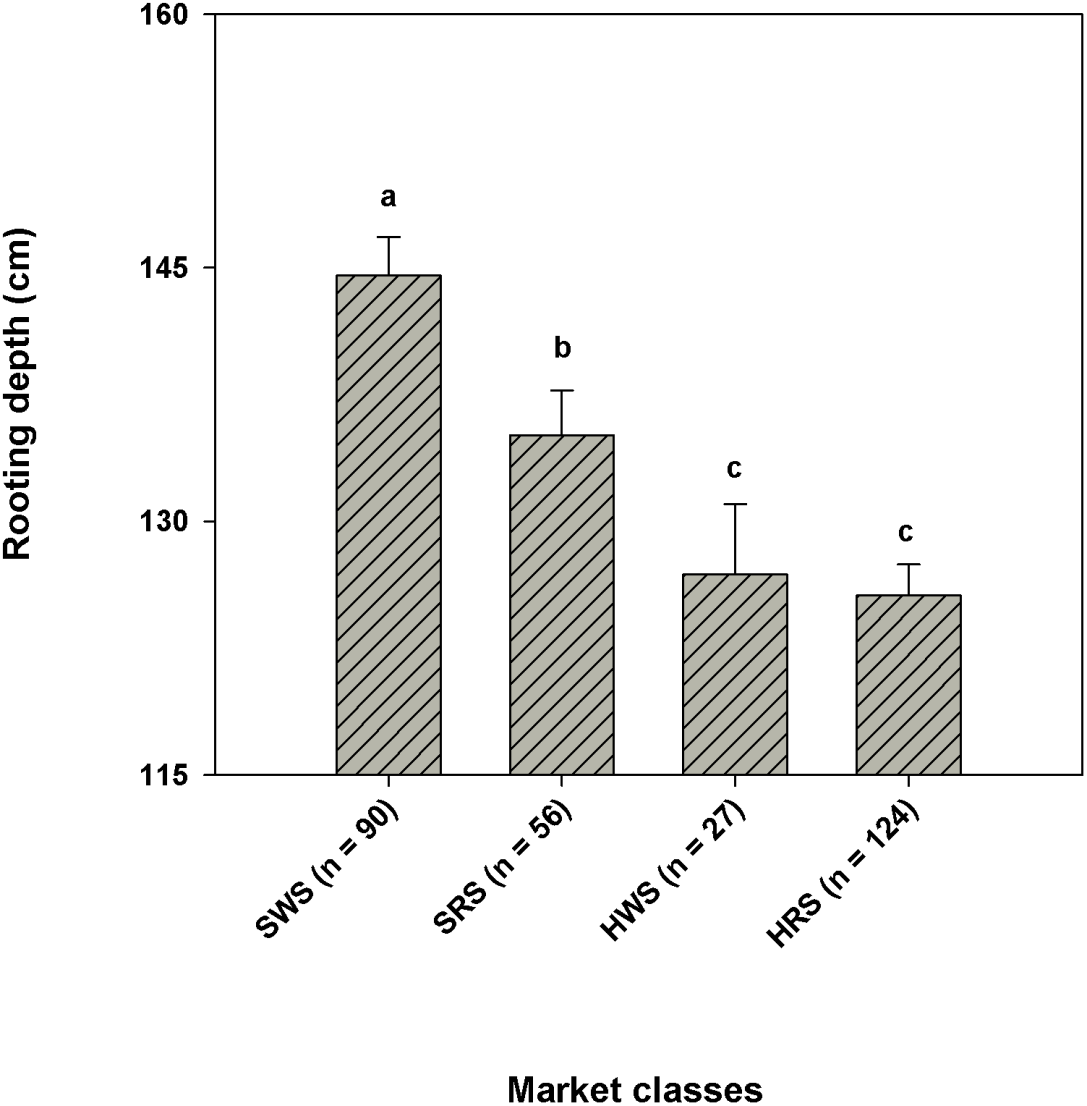
Rooting depth of spring wheat genotypes belonging to different market classes within the Cultivated Wheat Collection. Error bars represent standard errors. SWS – soft white spring; SRS – soft red spring; HWS – hard white spring; HRS – hard red spring.

The CWC germplasm included genotypes originating from 27 different countries. Country of origin had significant effect on rooting depth (P<0.05). When genotypes in the CWC germplasm were categorized into eight regional groups based on their country of origin, significant difference in the mean rooting depth was observed among the eight regions (P<0.05; Fig. 6A). The wheat genotypes collected from Australia, Mediterranean, and west Asia regions had greater rooting depth than those collected from south Asia, Latin America, Mexico, and Canada. West Asia (Fig. 6A) included Armenia (one genotype), Turkey (three genotypes), Iraq (one genotype), and Jordan (one genotype), which encompass the region where wheat originated. This shows that the six genotypes collected from the center of origin of wheat had deep root systems. When genotypes from USA were classified into 10 groups based on their state of origin, there was not much variation in the mean rooting depth among different groups (Fig. 6C). However, genotypes from Oregon had greater rooting depth than genotypes from other states such as North Dakota, Colorado, Arizona, and Minnesota. When genotypes in the CWC germplasm were categorized into four different market classes, they differed in rooting depth (P<0.0001). Soft wheat had greater rooting depth than hard wheat (Fig. 7). Soft white spring wheat had the largest rooting depth among the market classes evaluated in this research.

## Discussion

Considerable genetic variability was observed for root traits in the CWC germplasm or its subset. The extent of genetic variability is indicated by the large range observed for root traits. Because roots followed a zigzag pattern of growth within the columns, in many cases rooting depth attained values that exceeded column height. The P>0.05 for genotype-by-year interaction (Table 1) implies that genotypes had similar responses in both years for root traits. Plants were at flag leaf, booting, spike emergence, or flowering stages at the time of harvest. However, data analysis showed that except on plant height and tiller number, growth stage had no effect (P>0.05) on any of the root and shoot traits measured on the 297 genotypes in the CWC germplasm (data not shown).

Genotypes Sonora and Currawa were ranked high and genotype Vandal was ranked low for most root traits in the CWC germplasm or its subset. The contrasting genotypes for root traits identified in this study (Table 2, 3) offer useful plant materials that can be included in wheat improvement programs. Genotypes Federation 67 and McVEY were ranked in the lowest one third of genotypes for average root diameter and in the top one third of genotypes for root length and RLD in the upper soil profile (0–30 cm; Table 3). Decreased root penetration due to decreased root diameter [9] in genotypes Federation 67 and McVEY might have resulted in increased spreading behavior, which was manifested in terms of increased root length and RLD in the upper soil profile. Small root diameter and xylem vessels can enhance grain yield in wheat under terminal drought stress conditions because these traits help to conserve sufficient soil water for grain filling stage [5], [27].

In the present research, total root surface area showed a positive correlation with tiller number and shoot dry weight (Fig. 4). Previous reports in other cereals have suggested that water and nutrient uptake from the soil is proportional to contact area between root surface and soil [28], [29]. This indicates that resource uptake increases with root surface area. The increased resource uptake through increased root surface area might have helped the plant to produce more tillers. The increased tiller number, which leads to increased shoot biomass production, might be the reason for increased shoot dry weight.

The positive correlation between root dry weight and shoot dry weight (R^2^ = 0.41; Fig. 3A) observed in this research is consistent with reports on other crops [25], [30]. The increased resource capture achieved through increased root mass might have contributed to the increased shoot dry weight. In turn, the surplus of photoassimilates as a result of increased shoot growth might be allocated to roots that increased root dry weight. However, the amount of resource uptake by different genotypes was not quantified in this study to evaluate its effects on root and shoot dry weights.

The absence of correlation between plant height and root traits (Table 4, 5; Fig. 3B) observed in this research is supported by previous reports in wheat [31], chickpea [30], or field pea (*Pisum sativum* L.) [25]. It is reported in field pea that plant height is not expected to have a correlation with total root length and weight because total root length is determined by number and length of lateral roots [25]. Reports suggest that root length and weight are predominately controlled by different sets of genes compared to that of shoot length [31]. Some studies have reported that decreased plant height genes had no impact on root diameter [32], and root dry weight [33]. Even though plant height was not correlated with root traits and tiller number, a negative correlation was found between plant height and root: shoot ratio (Table 4, 5). This may be due to increased shoot biomass production and therefore, increased shoot dry weight by tall plants.

The positive relationship between coleoptile length and total root length (R^2^ = 0.43; Fig. 5) has important practical implications. Selection for a deep and prolific root system on the basis of total root length is not easy because it is difficult to measure roots in situ. In addition, direct selection for total root length is a destructive process and prevents selection. Therefore, nondestructive selection criteria for improved root traits are important. Because total root length and coleoptile length show a positive linear relationship, selecting genotypes with increased coleoptile length might result in genotypes with increased root length. Selection for coleoptile length is easy, non-destructive, and involves high heritability (h^2^>0.70) [34]. A long coleoptile enables sowing at greater soil depths where moisture is available [35], and improves seedling vigor and stand establishment [36].

**Figure 8:**
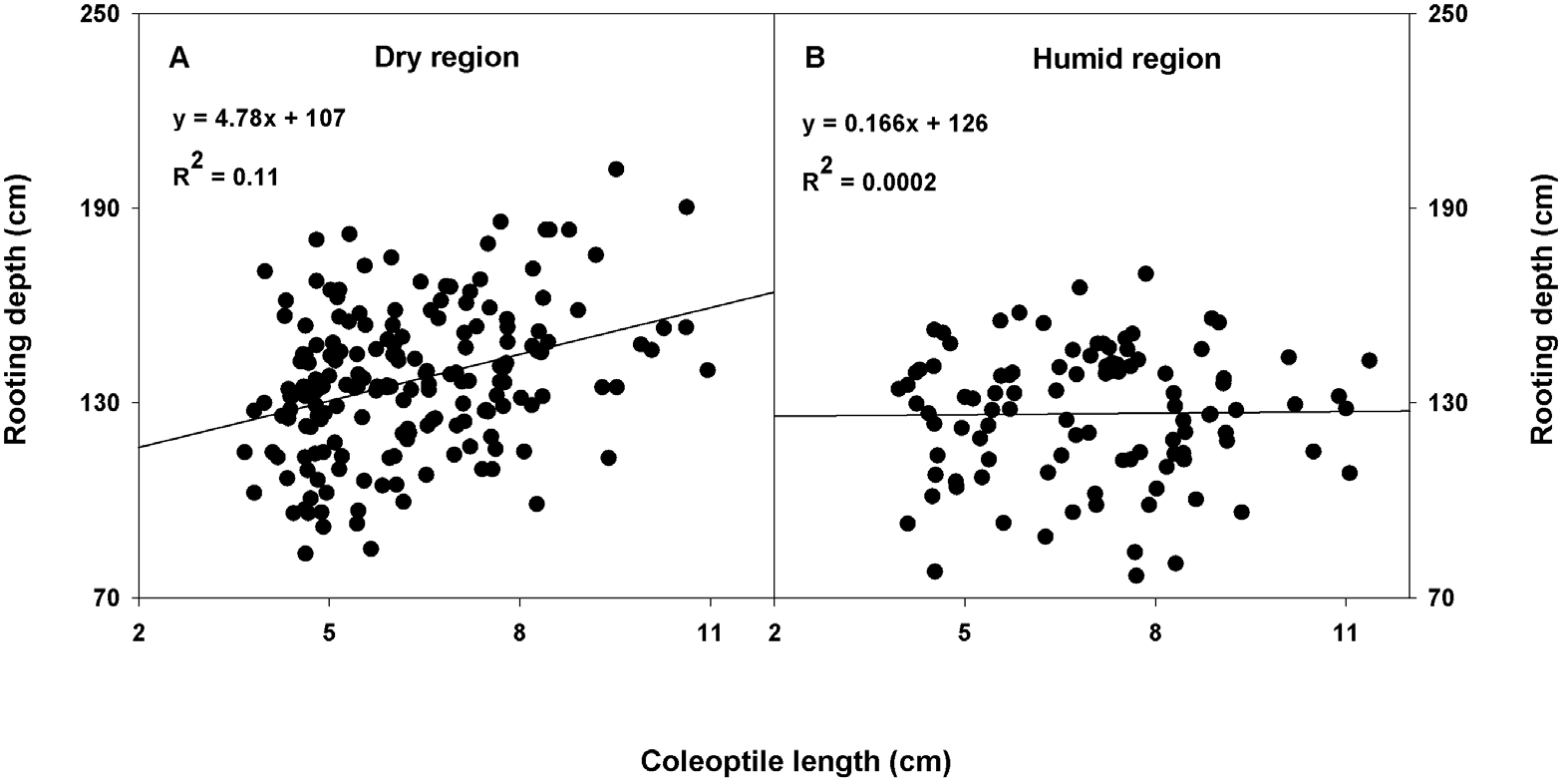
Relationship between coleoptile length and rooting depth among spring wheat genotypes originated from dry (n = 184) or humid regions (n = 104). Dry region included Argentina, Armenia, Australia, Chile, Egypt, Germany, Iraq, Japan, Jordan, Kenya, Lebanon, Libya, Mexico, Pakistan, South Africa, Turkey, and USA states of Arizona, California, Colorado, Idaho, Montana, Nebraska, Nevada, Oregon, Utah, and Washington. Similarly, humid region included Bangladesh, Brazil, Canada, Colombia, Guatemala, India, Nepal, Paraguay, Russia, Uruguay, and USA states of Indiana, Minnesota, North Dakota, Oklahoma, South Dakota, Vermont, and Wisconsin. Two genotypes with unknown country of origin and seven genotypes with unknown states of origin in the USA were not included in these figures.

When countries or USA states of origin of all 297 genotypes were broadly classified into dry or humid regions (Köppen-Geiger climate classification) [37], it had significant influence on the relationship between coleoptile length and rooting depth (P<0.05 for the effect of ‘coleoptile length-by-region’ interaction on rooting depth). Coleoptile length and rooting depth had a positive linear relationship with R^2^ = 0.11, in the dry region (Fig. 8). This implies that rooting depth increases with coleoptile length in the dry regions. Deep roots increase soil water extraction from deep soil layers where moisture is available [5] and longer coleoptiles improve stand establishment and vigor in deep-sown crop in the dry areas [36]. Therefore, both of these traits provide adaptational advantages to genotypes grown under soil moisture limited environments.

**Figure 9:**
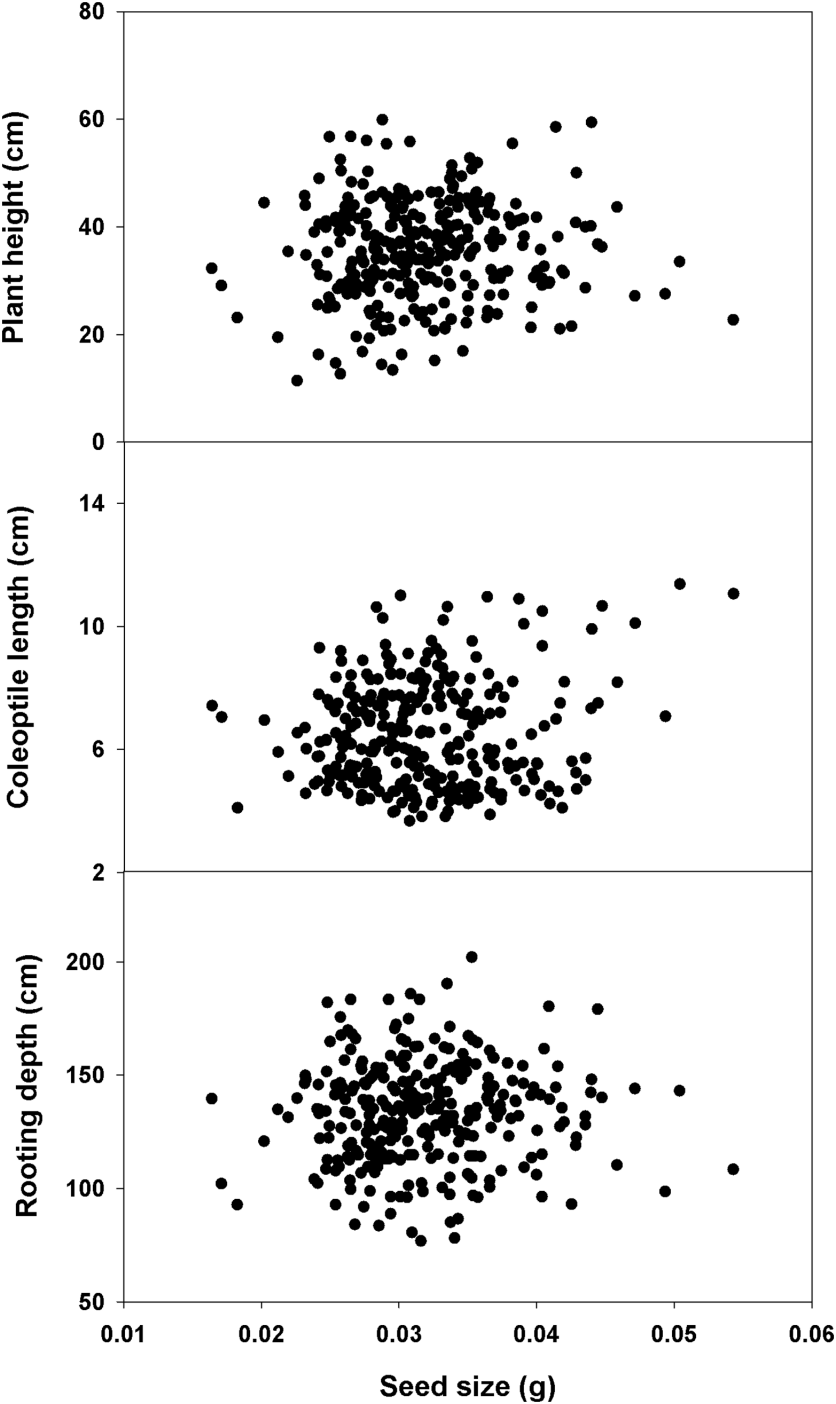
Relationship of seed size with plant height, coleoptile length, and rooting depth of 297 spring wheat genotypes of the Cultivated Wheat Collection.

None of the root traits evaluated in this research showed a correlation with seed size within the CWC germplasm or its subset (Table 4, 5). Slope was not significant when plant height, coleoptile length, and rooting depth were regressed against seed size within the CWC germplasm (Fig. 9). This shows that seeds with increased size may not always produce longer coleoptile, deeper roots, or taller plants. This result is in agreement with previous reports suggesting that seed size has no influence on coleoptile length and seedling emergence in wheat [24], [38], [39], [40]. However, contradictory reports also exist in literature that suggested a positive correlation between seed size and coleoptile length [41], [42], [43].

This research found that the geographic regions from which wheat genotypes originated had significant impacts on rooting depth (Fig. 6A). The wheat genotypes collected from Australia, Mediterranean, and west Asia regions possessed greater rooting depth compared with those collected from south Asia, Latin America, Mexico, and Canada. However, we acknowledge that different geographic regions were not represented by equal number of genotypes. Genotypes originated from dry regions had greater rooting depth than those from humid regions (Fig. 6B, D). Growth environments for wheat in Mediterranean, Australia, and west Asia regions are much drier than they are in the other regions such as south Asia, Latin America, or Canada [22], [44]. Maximum utilization of stored soil moisture is important for the dry environments in Australia, Mediterranean or west Asia regions [2]. Plant root systems in these regions are adapted to thrive on the available soil moisture and not deplete it before maturity [44]. Wheat depending on stored soil moisture needs a root system that reaches the deep soil profile [5], [45]. Thus, wheat genotypes that evolved in those drier areas might have adapted by increasing rooting depth to capture water from the deeper layers of soil. The wheat genotypes collected from Australia also had larger root diameter (mean ± SD, 1.1±0.31 mm) than those collected from other regions. Large diameter is an important trait of plant roots that helps them to penetrate deep soil layers, which is evident from the positive correlation between average root diameter and rooting depth in the present study (Table 5). Even though mean rooting depth did not show much variation among different states within the USA, a gradual decrease in rooting depth was noticed from west to east (Fig. 6C). This could be associated with general trends of increasing precipitation and decreasing temperature from west to east in the USA. The increased rooting depth may be an adpational trait of genotypes grown in comparatively drier areas of western USA to improve water absorption. We also observed differences in rooting depth among different market classes of wheat (Fig. 7). The greater rooting depth of soft wheat compared with hard wheat may be an inherent characteristic of soft wheat genotypes.

In summary, significant genetic variability was observed for root traits in the CWC germplasm or its subset. Genotypes Sonora and Currawa were ranked high and genotype Vandal was ranked low for most root traits. A strong positive relationship (R^2^≥0.35) was found between (1) root dry weight and shoot dry weight within the CWC germplasm and between total root surface area and tiller number; total root surface area and shoot dry weight; and total root length and coleoptile length within the subset. There was no correlation between plant height and most root traits within the CWC germplasm or its subset. Region of origin of wheat genotypes had significant impact on rooting depth in the CWC germplasm. The wheat genotypes collected from Australia, Mediterranean, and west Asia regions had greater rooting depth than those collected from south Asia, Latin America, Mexico, and Canada. Rooting depth differed among market classes of wheat genotypes in the CWC germplasm. Soft wheat had greater rooting depth than hard wheat in the CWC germplasm. The genetic variability identified in this research for root traits can be exploited to improve drought tolerance and/or resource capture in wheat. Our future research will evaluate drought tolerance of the contrasting genotypes identified in this study for root traits under controlled environment and field conditions.

## Supporting Information

Table S1 **Names, countries of origin, and market classes of 297 spring wheat genotypes of the Cultivated Wheat Collection.**
(DOCX)
