# Supplementary material for: Variability of Root Traits in Spring Wheat Germplasm
Source: PLoS One. 2014 Jun 19;9(6):e100317. doi: 10.1371/journal.pone.0100317 (PMC4063797; doi:10.1371/journal.pone.0100317)
Supplement: Table S1 — Names, countries of origin, and market classes of 297 spring wheat genotypes of the Cultivated Wheat Collection. (DOCX) [file pone.0100317.s001.docx]

Table S1. Names, countries of origin, and market classes of 297 spring wheat genotypes of the Cultivated Wheat Collection.

| Number | Genotype | Country of origin | Market class* |
| --- | --- | --- | --- |
| 1 | CWI50568 | Argentina | HRS |
| 2 | Marcos Juarez Inta | Argentina | HRS |
| 3 | Granero Inta | Argentina | HRS |
| 4 | Prointa Oasis | Argentina | HRS |
| 5 | Klein Dragon | Argentina | HWS |
| 6 | Pointa Federal | Argentina | SWS |
| 7 | Vanna | Arizona, USA | SWS |
| 8 | Westbred 906R | Arizona, USA | HRS |
| 9 | Westbred 911 | Arizona, USA | HRS |
| 10 | Westbred Express | Arizona, USA | HRS |
| 11 | AIM | Arizona, USA | HRS |
| 12 | Galgalos | Armenia | SWS |
| 13 | Hartog | Australia | HWS |
| 14 | Federation | Australia | SWS |
| 15 | Currawa | Australia | SWS |
| 16 | Hard Federation | Australia | HWS |
| 17 | White Federation | Australia | HWS |
| 18 | Bunyip | Australia | SWS |
| 19 | Florence Aka Quality | Australia | SWS |
| 20 | Major | Australia | SWS |
| 21 | Onas | Australia | SWS |
| 22 | BAW898 | Bangladesh | HWS |
| 23 | BR 18 | Brazil | HRS |
| 24 | Ramona | California, USA | SWS |
| 25 | Ramona 50 | California, USA | HRS |
| 26 | Onas 53 | California, USA | SWS |
| 27 | Pacific Bluestem 37 | California, USA | SWS |
| 28 | Awned Onas | California, USA | SWS |
| 29 | Baart Early Selection | California, USA | SRS |
| 30 | Canadian Red | California, USA | HWS |
| 31 | Baart 46 | California, USA | SWS |
| 32 | Pilcraw | California, USA | SWS |
| 33 | Early Baart | California, USA | SWS |
| 34 | Red River 68 | California, USA | HRS |
| 35 | Kenhi | Canada | SWS |
| 36 | Selkirk | Canada | HRS |
| 37 | Redman | Canada | HRS |
| 38 | Canthatch | Canada | HRS |
| 39 | Regent | Canada | HRS |
| 40 | Saunders | Canada | HRS |
| 41 | Ruby | Canada | HRS |
| 42 | Reward | Canada | HRS |
| 43 | Preston | Canada | HRS |
| 44 | Supreme | Canada | HRS |
| 45 | Rescue | Canada | HRS |
| 46 | Red Bobs | Canada | HRS |
| 47 | Manitou | Canada | HRS |
| 48 | Marquis | Canada | HRS |
| 49 | Millaleau Inia | Chile | HWS |
| 50 | Orofen 60 | Chile | SRS |
| 51 | Narino 59 | Colombia | SWS |
| 52 | Crespo | Colombia | SRS |
| 53 | Ica Yacuanquer | Colombia | SWS |
| 54 | Saxon | Colorado, USA | HRS |
| 55 | Bounty 208 | Colorado, USA | HRS |
| 56 | Sea Island | Colorado, USA | HRS |
| 57 | Gypsum | Colorado, USA | SWS |
| 58 | Bounty 309 | Colorado, USA | HRS |
| 59 | Sakha 8 | Egypt | SWS |
| 60 | Sakha 69 | Egypt | HWS |
| 61 | Schlanstedt | Germany | SRS |
| 62 | Maya 74 | Guatemala | HWS |
| 63 | Pomerelle | Idaho, USA | SWS |
| 64 | 64Ab9405 | Idaho, USA | SWS |
| 65 | Idaho 61M3404 | Idaho, USA | SWS |
| 66 | Idaho 62M9-224 | Idaho, USA | SWS |
| 67 | Twin | Idaho, USA | SWS |
| 68 | Owens | Idaho, USA | SWS |
| 69 | IDO190 | Idaho, USA | SWS |
| 70 | IDO232 | Idaho, USA | SWS |
| 71 | Copper | Idaho, USA | HRS |
| 72 | Vandal | Idaho, USA | HRS |
| 73 | Idaho 266 | Idaho, USA | SWS |
| 74 | Whitebird | Idaho, USA | SWS |
| 75 | Alturas | Idaho, USA | SWS |
| 76 | Jubilee | Idaho, USA | SWS |
| 77 | Jefferson | Idaho, USA | HRS |
| 78 | Centennial | Idaho, USA | SWS |
| 79 | Peak | Idaho, USA | HRS |
| 80 | Peak 72 | Idaho, USA | HRS |
| 81 | Sterling | Idaho, USA | SWS |
| 82 | Irridur | Idaho, USA | HRS |
| 83 | Borah | Idaho, USA | HRS |
| 84 | Treasure | Idaho, USA | SWS |
| 85 | Bliss | Idaho, USA | SWS |
| 86 | Federation 67 | Idaho, USA | SWS |
| 87 | Idaed | Idaho, USA | SWS |
| 88 | Indian | Idaho, USA | SWS |
| 89 | Springfield | Idaho, USA | SWS |
| 90 | Fielder | Idaho, USA | SWS |
| 91 | McKay | Idaho, USA | HRS |
| 92 | Moran | Idaho, USA | SWS |
| 93 | Fieldwin | Idaho, USA | SWS |
| 94 | Lemhi 66 | Idaho, USA | SWS |
| 95 | Lolo | Idaho, USA | HWS |
| 96 | Lemhi | Idaho, USA | SWS |
| 97 | Lemhi 53 | Idaho, USA | SWS |
| 98 | Kalyansona | India | HWS |
| 99 | Safed Lerma | India | SWS |
| 100 | Sonalika | India | SWS |
| 101 | UP301 | India | SWS |
| 102 | PBW343 | India | HWS |
| 103 | BW21695 | India | SWS |
| 104 | FREX | Indiana, USA | SRS |
| 105 | Abu Ghraib#3 | Iraq | HWS |
| 106 | White Fife | Japan | HWS |
| 107 | Nesser | Jordan | HWS |
| 108 | Kenya Kwale | Kenya | SRS |
| 109 | Cham 6 | Lebanon | HWS |
| 110 | Arz | Libya | HWS |
| 111 | Yecora Rojo | Mexico | HRS |
| 112 | Sonora | Mexico | SWS |
| 113 | Gabo 60 | Mexico | HWS |
| 114 | Nacozari F 76 | Mexico | HWS |
| 115 | Bluebird 15 | Mexico | HWS |
| 116 | San Cayetano S 97 | Mexico | HWS |
| 117 | Temporalera M87 | Mexico | HRS |
| 118 | Tinamou Ii | Mexico | HRS |
| 119 | Arivechi M 92 | Mexico | HRS |
| 120 | Yaqui 50 | Mexico | HRS |
| 121 | Pitic 62 | Mexico | SRS |
| 122 | Nadadores M 63 | Mexico | HRS |
| 123 | Sonora 64 | Mexico | HRS |
| 124 | Inia F66 | Mexico | HRS |
| 125 | Bajio | Mexico | HRS |
| 126 | Calidad | Mexico | HRS |
| 127 | Potam S 70 | Mexico | HRS |
| 128 | Tanori F 71 | Mexico | SRS |
| 129 | Jupateco F 73 | Mexico | HRS |
| 130 | Salamanca 75 | Mexico | SRS |
| 131 | Pavon F 76 | Mexico | HWS |
| 132 | Hermosillo M77 | Mexico | HRS |
| 133 | Seri M 82 | Mexico | HWS |
| 134 | Rayon F 89 | Mexico | HWS |
| 135 | TIA.1 | Mexico | HWS |
| 136 | Borlaug M 95 | Mexico | HRS |
| 137 | Inifap M 97 | Mexico | HRS |
| 138 | Tobarito M 97 | Mexico | HRS |
| 139 | Andes-56 | Mexico | SRS |
| 140 | Lerma Rojo 64 | Mexico | SRS |
| 141 | V-17 | Mexico | HRS |
| 142 | II-53-521 | Minnesota, USA | HRS |
| 143 | Chris | Minnesota, USA | HRS |
| 144 | II-55-1 | Minnesota, USA | HRS |
| 145 | II-58-60 | Minnesota, USA | HRS |
| 146 | II-62-78 | Minnesota, USA | HRS |
| 147 | Wheaton | Minnesota, USA | HRS |
| 148 | II-64-20 | Minnesota, USA | HRS |
| 149 | Vance | Minnesota, USA | HRS |
| 150 | Verde | Minnesota, USA | HRS |
| 151 | Prospur | Minnesota, USA | HRS |
| 152 | White Marquis | Minnesota, USA | HWS |
| 153 | Thatcher | Minnesota, USA | HRS |
| 154 | Era | Minnesota, USA | HRS |
| 155 | Produra | Minnesota, USA | HRS |
| 156 | MN 6616M | Minnesota, USA | HRS |
| 157 | MN 6898 | Minnesota, USA | HRS |
| 158 | Norm | Minnesota, USA | HRS |
| 159 | Lee | Minnesota, USA | HRS |
| 160 | Kitt | Minnesota, USA | HRS |
| 161 | Newana | Montana, USA | HRS |
| 162 | Pondera | Montana, USA | HRS |
| 163 | Norana | Montana, USA | HRS |
| 164 | Kahla | Montana, USA | HRS |
| 165 | Challis | Montana, USA | SWS |
| 166 | Centana | Montana, USA | HRS |
| 167 | Probrand 751 | Nebrasca, USA | HRS |
| 168 | McVEY | Nebrasca, USA | HRS |
| 169 | Klasic | Nebrasca, USA | HWS |
| 170 | Annapurna 1 | Nepal | HWS |
| 171 | UP262 | Nepal | HWS |
| 172 | New Zealand | Nevada, USA | SWS |
| 173 | Mida | North Dakota, USA | SRS |
| 174 | Conley | North Dakota, USA | HRS |
| 175 | Premier | North Dakota, USA | SRS |
| 176 | Cadet | North Dakota, USA | SRS |
| 177 | Ceres | North Dakota, USA | HRS |
| 178 | Chinook | North Dakota, USA | HRS |
| 179 | Rolette | North Dakota, USA | HWS |
| 180 | Rival | North Dakota, USA | HRS |
| 181 | Justin | North Dakota, USA | HRS |
| 182 | Leeds | North Dakota, USA | HRS |
| 183 | ND 59-120A | North Dakota, USA | HRS |
| 184 | ND 407 | North Dakota, USA | HRS |
| 185 | Waldron | North Dakota, USA | HRS |
| 186 | ND 66 | North Dakota, USA | SRS |
| 187 | CI014952 | North Dakota, USA | HRS |
| 188 | CI014953 | North Dakota, USA | HRS |
| 189 | D 6647 | North Dakota, USA | HWS |
| 190 | ND 467 | North Dakota, USA | HRS |
| 191 | ND 476 | North Dakota, USA | HRS |
| 192 | Ellar | North Dakota, USA | HRS |
| 193 | Edmore | North Dakota, USA | HWS |
| 194 | Coteau | North Dakota, USA | HRS |
| 195 | D804 | North Dakota, USA | HWS |
| 196 | D7925 | North Dakota, USA | HWS |
| 197 | Amidon | North Dakota, USA | HRS |
| 198 | Pierce | North Dakota, USA | HWS |
| 199 | Olaf | North Dakota, USA | HRS |
| 200 | Ward | North Dakota, USA | HWS |
| 201 | Pilot | North Dakota, USA | HRS |
| 202 | Sentry | North Dakota, USA | HWS |
| 203 | Langdon | North Dakota, USA | HWS |
| 204 | Wells | North Dakota, USA | HWS |
| 205 | ND 202-2 | North Dakota, USA | HRS |
| 206 | ND 271 | North Dakota, USA | HRS |
| 207 | ND 229-1 | North Dakota, USA | HRS |
| 208 | ND 287 | North Dakota, USA | HRS |
| 209 | ND 22 | North Dakota, USA | SRS |
| 210 | Monroe | North Dakota, USA | HWS |
| 211 | ND 13-137 | North Dakota, USA | HRS |
| 212 | ND 2710 | North Dakota, USA | HRS |
| 213 | Fortuna | North Dakota, USA | HRS |
| 214 | PJ62/GB55 | North Dakota, USA | HWS |
| 215 | STW 598874 | Oklahoma, USA | HRS |
| 216 | YSCA-1 | Oklahoma, USA | SRS |
| 217 | Union | Oregon, USA | SWS |
| 218 | Reliance | Oregon, USA | HRS |
| 219 | Oregon Zimmerman | Oregon, USA | SWS |
| 220 | Pacific Bluestem | Oregon, USA | SWS |
| 221 | Bluechaff | Oregon, USA | SWS |
| 222 | Big Club | Oregon, USA | SWS |
| 223 | Hard Federation (-31) | Oregon, USA | HWS |
| 224 | Beaver | Oregon, USA | SWS |
| 225 | Wilbur | Oregon, USA | SWS |
| 226 | Winsome | Oregon, USA | HWS |
| 227 | Kinney | Oregon, USA | SRS |
| 228 | Mexipak 65 | Pakistan | HWS |
| 229 | Faislabad 83 | Pakistan | HWS |
| 230 | Punjab 88 | Pakistan | HWS |
| 231 | Bahawalpur 79 | Pakistan | HWS |
| 232 | Pirsabak 85 | Pakistan | HWS |
| 233 | Sariab-92 | Pakistan | SWS |
| 234 | Zamindar 80 | Pakistan | HWS |
| 235 | Pakistan 81 | Pakistan | HWS |
| 236 | Itapua 40-Obligado | Paraguay | SRS |
| 237 | Ian 8-Pirapo | Paraguay | HRS |
| 238 | Pavon | Paraguay | HRS |
| 239 | Cordillera 3 | Paraguay | HRS |
| 240 | Kubanka | Russia | HRS |
| 241 | Lagoda | Russia | HRS |
| 242 | Liesbeck | South Africa | SRS |
| 243 | Rushmore | South Dakota, USA | HRS |
| 244 | Spinkcota | South Dakota, USA | SRS |
| 245 | Hope | South Dakota, USA | SRS |
| 246 | Penjamo T 62 | Turkey | SRS |
| 247 | Gonen | Turkey | HWS |
| 248 | Cumhuriyet 75 | Turkey | HRS |
| 249 | WL 444 | Unknown | HRS |
| 250 | Chivito | Unknown | SWS |
| 251 | Estanzuela Pelon 90 | Uruguay | HRS |
| 252 | Munich | USA | HWS |
| 253 | Macon | USA | HWS |
| 254 | Little Club | USA | SWS |
| 255 | Rink | USA | SWS |
| 256 | Yecora Rojo 76 | USA | HRS |
| 257 | Bronze Chief | USA | HRS |
| 258 | Kodiak Dwarf | USA | HRS |
| 259 | Utac | Utah, USA | SRS |
| 260 | Touse | Utah, USA | SWS |
| 261 | Dicklow | Utah, USA | SWS |
| 262 | Surprise | Vermont, USA | SWS |
| 263 | Marfed | Washington, USA | SWS |
| 264 | Sel 90 | Washington, USA | SWS |
| 265 | WA 6101 | Washington, USA | SWS |
| 266 | WA 7175 | Washington, USA | SWS |
| 267 | Spillman | Washington, USA | HRS |
| 268 | ARS95 451 | Washington, USA | SWS |
| 269 | ARS95 457 | Washington, USA | SWS |
| 270 | Eden | Washington, USA | SWS |
| 271 | Alpowa | Washington, USA | SWS |
| 272 | Edwall | Washington, USA | SWS |
| 273 | Penawawa | Washington, USA | SWS |
| 274 | Tara 2002 | Washington, USA | HRS |
| 275 | Scarlet | Washington, USA | HRS |
| 276 | Calorwa | Washington, USA | SWS |
| 277 | Zak | Washington, USA | SWS |
| 278 | Wawawai | Washington, USA | SWS |
| 279 | IDO377s | Washington, USA | HWS |
| 280 | Urquie | Washington, USA | SWS |
| 281 | Redchaff | Washington, USA | SWS |
| 282 | Wadual | Washington, USA | SWS |
| 283 | Wakanz | Washington, USA | SWS |
| 284 | Wampum | Washington, USA | HRS |
| 285 | Walladay | Washington, USA | SWS |
| 286 | Waid | Washington, USA | HWS |
| 287 | Waverly | Washington, USA | SWS |
| 288 | Orfed | Washington, USA | SWS |
| 289 | Allen | Washington, USA | HRS |
| 290 | Hybrid 63 | Washington, USA | HWS |
| 291 | Hybrid 143 | Washington, USA | SWS |
| 292 | Hybrid 123 | Washington, USA | SRS |
| 293 | Hyper | Washington, USA | SWS |
| 294 | Wandell | Washington, USA | HRS |
| 295 | Hollis | Washington, USA | HRS |
| 296 | Flomar | Washington, USA | HWS |
| 297 | Henry | Wisconsin, USA | SRS |

*soft white spring (SWS); soft red spring (SRS); hard white spring (HWS); and hard red spring (HRS).
